# Draft Genome Sequence for the Type Strain *Vulcanibacillus modesticaldus* BR, a Strictly Anaerobic, Moderately Thermophilic, and Nitrate-Reducing Bacterium Isolated from Deep-Sea Hydrothermal Vents of the Mid-Atlantic Ridge

**DOI:** 10.1128/genomeA.01246-16

**Published:** 2016-11-10

**Authors:** Christopher A. Abin, James T. Hollibaugh

**Affiliations:** a Department of Microbiology, University of Georgia, Athens, Georgia, USA; b Department of Marine Sciences, University of Georgia, Athens, Georgia, USA

## Abstract

*Vulcanibacillus modesticaldus* BR^T^ was isolated from calcite-rich, metalliferous core samples collected at the Rainbow deep-sea hydrothermal vent field on the Mid-Atlantic Ridge. Here, we report the 2.2-Mb draft genome sequence for this strain, consisting of 100 contigs with a G+C content of 33.6% and 2,227 protein-coding sequences.

## GENOME ANNOUNCEMENT

*Vulcanibacillus modesticaldus* BR^T^ was isolated from core samples collected at the Rainbow hydrothermal vent field on the Mid-Atlantic Ridge (MAR) (36°14′ N, 33°54′ W) (1). The Rainbow vent field is located at a depth of ~2,300 m on the western flank of a nonvolcanic ridge of the MAR, southwest of the Azores Archipelago (2). Active chimneys emit acidic (pH 2.8), high-temperature (365°C) fluids enriched in H_2_, CO_2_, CO, CH_4_, metals, and rare earth elements (3, 4). *V. modesticaldus* BR^T^ was able to grow at temperatures from 37 to 60°C, pH values of 6.0 to 8.5, and salinities of 1 to 4%. The strain grew chemoheterotrophically with organic acids, carbohydrates, and complex proteinaceous substrates as electron donors with nitrate as the sole electron acceptor. Nitrite produced from dissimilatory nitrate reduction is not reduced further to ammonium or to N_2_, indicating that the strain is unable to perform dissimilatory nitrate reduction to ammonium (DNRA) or denitrification (1).

Pure genomic DNA from *V. modesticaldus* BR^T^ was obtained from the Leibniz Institute German Collection of Microorganisms and Cell Cultures (DSMZ, Braunschweig, Germany) and quantified using a Qubit fluorometer (Thermo-Fisher Scientific/Life Technologies, Waltham, MA, USA). Genome sequencing was performed using the MiSeq platform (Illumina, San Diego, CA, USA) with 250-bp paired-end reads. A total of 2,728,778 reads were generated, providing about 287-fold median coverage of the genome. The sequence processing toolkit seqtk version 1.0-r63 (https://github.com/lh3/seqtk) was used to randomly subsample the reads to an approximate 85-fold median coverage. Adapter removal, quality trimming, error correction, and *de novo* assembly were performed using the A5-miseq pipeline (5). The assembly yielded 100 contigs with a total genome size of 2,224,341 bp. The maximum and *N*_50_ contig sizes were 161,986 bp and 50,162 bp, respectively. The G+C content was 33.6%, a value slightly lower than the 34.5% previously obtained by reversed-phase high-pressure liquid chromatography (1). Genome annotation was performed by the Rapid Annotations using Subsystems Technology (RAST) server (6). A total of 2,227 protein-coding sequences and 64 tRNA genes were predicted. Fifty-two percent (1,156) of the coding sequences were assigned to subsystems. Phylogenomic analysis by AMPHORA2 (7) was used to estimate genome completeness. All 31 phylogenetic marker genes essential in bacteria were found to be present in the draft genome.

The genome of *V. modesticaldus* BR^T^ encodes multiple pathways involved in the metabolism of carbohydrates and organic acids. The full assortment of genes required for glycolysis and the tricarboxylic acid cycle is present, which is consistent with the ability of the strain to grow using acetate, pyruvate, and several mono- and polysaccharides as electron donors. The genome also contains an operon encoding a membrane-bound dissimilatory nitrate reductase (Nar), as well as two nitrate-nitrite transporters. The knowledge obtained from this draft genome will help shed light on the evolution and physiology of moderately thermophilic bacteria inhabiting deep-sea hydrothermal vent ecosystems.

### Accession number(s).

This whole-genome shotgun project has been deposited at DDBJ/ENA/GenBank under the accession number MIJF00000000. The version described in this paper is the first version, MIJF01000000.
